# Ovariotomy—Report of a Case of Successful Removal of a Multilocular Ovarian Tumor, Weighing Twenty Pounds; the Pedicle Returned into the Abdominal Cavity, the Ligatures Being Applied to the Vessels Separately and Cut Closely

**Published:** 1866-06

**Authors:** J. F. Miner


BUFFALO

### Medical and Surgical Journal.

VOL. V. JUNE, 1866. No. 11.

ART. I.—*Ovariotomy—Report of a case of successful removal of a Multilocular Ovarian Tumor, weighing twenty pounds; the pedicle returned into the abdominal cavity, the ligatures being applied to the vessels separately and cut closely.* BY J. F. MINER, M. D.

Operation for removal of ovarian disease, is not now by any means very rare, and the following case is not published with this view, but rather to make one additional report to the number of successful operations, which, in the aggregate, is not yet very great, and also to illustrate one or two points of practical importance in the treatment of similar cases.

*Mrs. James McGowan*, aged 25 years, consulted me first in the spring of 1865. She had been married two years; was regular in menstruation; had never been pregnant; was in good health. In the left ovarian region was a distinct, movable, not very tender tumor, about six inches in diameter, attended by considerable pain, which was the first symptom and what first attracted her attention, and was present and troublesome for some time before the tumor was noticed. She had consulted a good many physicians. *Dr. Z. Pitcher*, of Detroit, and perhaps others had correctly diagnosed the disease, and recommended removal of the tumor as the only plan of radical cure. Explanation of the nature of the disease and the danger of attempting removal was faithfully and fully made, and patient left to consider the hopes it offered of recovery and the fearful chances of early fatal termination. These visits were repeated at short intervals for more than a year, when, with great firmness and determination, she appointed her time for operation, saying that life was no longer worth having in such pain and misery. When she had thus decided she was encouraged, and the most cheerful and hopeful view consistent with the nature of the case was given her, believing that despondency and gloom were actively depressing agents, while hope and confidence would prove most powerful tonics.

The tumor had now greatly enlarged, so as to interfere with respiration and to cause much distress, which no doubt would have been temporarily relieved by tapping. The general health was becoming impaired, the expression was care-worn and very anxious. That the tumor was ovarian appeared quite certain, yet the fluid portion was so great and ascites so closely imitated in the general appearance, that doubt as to the nature of the case was expressed by professional friends, who were invited to visit and examine the tumor with me. The history, symptoms and appearance were to my mind so conclusive that no hesitation was allowed place, and great confidence entertained, though of course in absence of positive knowledge.

*April 3d*, 1866, in presence of, and assisted by, Drs. Lothrop, Eastman, Barnes, Mixer, Wyckoff, Nichell, M. H. Shaw, Frank King, Eugene Shaw, private pupils and a number of students, the operation was made in the following manner:—The patient being fully under the influence of chloroform division was made in the usual manner, about three inches in length down to the peritoneum. A trocar and canula was introduced and a small quantity of serum drawn from the abdominal cavity; the peritoneum was then divided upon a director and the tumor exposed. Examination showed but few adhesions, which were separated with the hand, except in one instance in which a portion of adherent omentum was divided with ecraseur. A trocar and canula was now introduced into a cyst of the tumor and about four quarts of a thick honey-like fluid evacuated; after which other cysts were evacuated in similar manner, until the fluid portion of the tumor had been removed, as fully as possible, traction being all the time made upon the mass, with the view to prevent escape of any of its contents into the abdominal cavity and to obtain its elevation, sufficient to admit of the division of the pedicle. By this means the mass, which in the aggregate weighed twenty pounds, the solid portion being about five and a half, and the remainder fluid, was extracted from the abdominal cavity through an incision three and a half inches in extent. By this process there was no exposure of intestine at any time and very little of omentum. The pedicle was found not large, and a double ligature passed and secured upon either side; the division was now made with the ecraseur, hoping thus to prevent hemorrhage from the smaller vessels. After removal of the tumor, the vessels of the pedicle, seven or eight in number, were *ligated separately*, the double ligature before mentioned being untied upon one side, and then the other, thus maintaining control of the pedicle, while all its bleeding vessels were carefully ligated, the larger ones with silver wire and some smaller ones with silk ligatures, all being cut closely, and the double ligature removed. After hemorrhage had ceased entirely the pedicle was allowed to return, and there having been no escape of blood or contents of the cysts into the abdominal cavity, no sponging was necessary, and the incision was closed. Five deep silver sutures were carefully introduced down accurately to the peritoneal membrane, but not through it. It was thus placed closely in apposition, and still no irritation from suture would be produced. A wetted compress was laid over the wound and bandage lightly applied. The operation thus briefly described occupied in all about two hours; was made in a small private room, kept at a temperature of about blood heat; and when completed the patient very soon rallied and appeared cheerful and hopeful as ever. One-half grain morphine was given subcutaneously, and same quantity ordered every four hours if in pain.

*April 4th.* Had slept some through the night, but suffered much from nausea and vomiting—food and medicine immediately ejected from the stomach. Pain had been mostly allayed by the anodyne. Pulse 120 per minute and strong. Abdomen tender on pressure, hard and a little swollen at the lower portion. Considerable thirst and no desire for food. Urine drawn, and instruction given the nurse so that she introduced the catheter drawing the urine every few hours during the twenty days succeeding the operation, when it was voided naturally. *Tinc. opii* was now prescribed in place of *morphia* with the view that the nausea and vomiting might be thus lessened; one-half teaspoonful every four hours, was continued during the period of convalescence.

*April 5th, 6th, and 7th*, the patient remained essentially the same. Vomiting and pain being very troublesome, little if any food retained; sleep imperfect and unrefreshing; thirst constant; heat of the surface of the body alternating with profuse perspiration.

*April 8th*, to the previous symptoms were now added a distinct, circumscribed hardness, occupying the left ovarian region. Pulse 135 per minute; vomiting lessening; milk punch and beef essence retained much better.

*April 9th.* Pain, tenderness and swelling in the left ovarian region increased. Pulse 145 per minute. Some delirium, great restlessness, countenance anxious, still without having had at any time distinct chill. Other symptoms about the same. Bowels moved by injection; urine drawn and opium continued as usual.

*April 10th.* During the night, pus had escaped in small quantity near the lower point of the incision made for removal of the tumor, the remainder of the incision being closed perfectly, some of the sutures having been previously removed. Pressure upon the ovarian region caused increased flow of pus. Pulse still remained rapid and irritable; vomiting troublesome; very little desire for food. Adhesive inflammation had protected the abdominal cavity, and provided a safe way of escape for the products of any reparative processes which might take place in the divided pedicle, which in this instance furnished a large quantity of healthy pus. From the time of the escape of the purulent accumulation, all the symptoms gradually abated, and upon the twenty-fourth day after the operation the patient commenced to attend to some of her household duties. Examination at the end of the seventh week shows still a barely perceptible discharge from the wound; the patient healthy and active, walking a mile or more without fatigue, and having gained greatly in flesh and strength.

A more detailed account of the appearances and symptoms might interest some who are watching anxiously the progress of similar cases, but, it is hoped that what has been given, is sufficiently descriptive of the case to afford a correct idea of the course pursued and of the chief peculiarities of an interesting instance of recovery, possessing some points of interest which are quite remarkable.

There are a few important practical points in the report of such cases, to which it is proper to allude. The results of ovariotomy depend upon the condition of the patient when the operation is made, the manner of making it and the after-treatment.

We shall take the liberty to copy from interesting statistics collated by *Dr. E. R. Peaslee* of New York, and published in the *American Journal of Medical Sciences* for January, 1865. His statistics are for the years 1860, '61, '62 and '63, including 150 cases which have been published during the four years. Mr. J. Clay of Birmingham, collected *all* the reported cases of ovariotomy up to the commencement of 1860, amounting to 425 completed operations which had been reported in all countries. Dr. Peaslee's collection is therefore a continuation of his, up to the beginning of 1864. Of Mr. Clay's 425 cases 57 per cent. recovered, and 43 per cent. terminated fatally. Dr. Peaslee's 150 cases 99, or 66 per cent. recovered, and 34, or 43 per cent. terminated fatally. The greater success of the operation for the four years of his collection he attributes to improvements in the operation itself, and more judicious after-treatment.

That Dr. Peaslee's cases do not represent accurately the rate of mortality after this operation may be regarded certain; that it is correct of the reported cases is doubtless true. But for very obvious reasons, surgeons are more ready to report successful than unfortunate cases, so that some unsuccessful operations were made but were not published; there were enough of these to have greatly augmented the proportion of fatal cases. The causes of death in the 51 fatal cases of ovariotomy collected by Dr. Peaslee were, peritonitis 12, septicæmia (pyæmia) 9, shock or collapse 7, exhaustion 7, hemorrhage 1, strangulation of intestine in incision 1, diarrhœa 1, erysipelas 1, tetanus 1, ulceration through the bladder 1, cause not stated 10.

The following statistics are copied from Dr. Peaslee's paper:

"1. *Influence of Age.*—The following table gives the ages in 116 of the cases here collated, and the results of the operation:

| | | |
|---|---|---|
| Under 20 years, | 8 cases. | 50 per cent. recovered. |
| 20 to 25 " | 16 " | 75 " " |
| 25 " 30 " | 13 " | 76.92 " " |
| 30 " 35 " | 24 " | 45.83 " " |
| 35 " 40 " | 20 " | 80 " " |

| | | |
|---|---|---|
| 40 to 45 years, | 10 cases. | 60 per cent. recovered. |
| 45 " 50 " | 7 " | 57.14 " " |
| 50 " 55 " | 10 " | 80 " " |
| 55 x " | 8 " | 85 " " |
| not stated | 34 " | |

Thus the most unfavorable age is under 20; next, from 30 to 35; and the average of the whole decade from 40 to 50 is but 58.82 per cent. of recoveries. From 30 to 35 is perhaps the period in which the effects of child-bearing are most exhaustingly felt, while that from 40—and especially from 45—to 50, is another critical season for women.

On the contrary, the highest per centage of recoveries occur in those above 55 years (85 per cent.;) and the average above the age of 50 is 83⅓ per cent. The average from 20 to 30 years is 75.85 per cent. The oldest patient on whom ovariotomy has yet been performed is Dr. Bennett's, of Danbury, Conn., her age being 75 years. She recovered.

2. *Married or Unmarried State.*—Of 116 cases in the preceding list, 64 were married, 52 were single.

Of the married, 38 or 59⅜ per cent. recovered.

" single, 38 or 73⅓ " "

3. *General Health at Time of Operation.*—Of 98 cases the general health was robust in 41 cases, impaired in 47 cases, and broken down in 10 cases.

Of those in robust health, 31 or 75.6 per cent. recovered.
" " impaired " 30 or 63.83 " "
" " broken " 4 or 40 " "

This is a point of great practical moment, and should be noted in all future reports. 52 cases out of the 150 are not represented in the above specifications at all; though they included 34 recoveries and 18 deaths. It is my conviction that the most favorable condition under this head is *slightly impaired* health.

4. *The Kind of Tumor.*—61 of the preceding cases were polycystic tumors, and 11 cases were monocystic.

Of the polycystic, 42 or 68.85 per cent. recovered.
" monocystic, 8 or 72.72 " "

Here again we need to include all the cases; since, as just shown, the recoveries in *both* kinds of tumors are above the general average of 66 per cent. of all operated upon.

5. *Size of the Tumor.*—If we arrange the tumors weighing less than 15 lbs., under the head of "small," and the rest of "large," we find that—

Of 6 cases of small tumor, 5 or 83⅓ per cent. recovered.
" 49 " large " 37 or 86.04 " "

The figures are here too high, as under the preceding head.

6. *Duration of Tumor after it became appreciable.*

Of 23 cases of tumor of less than 1½ year, 11 or 47.82 per cent. recovered.

" 41 " " 1½ year and over, 31 or 72.72 " "

A longer duration generally implies a greater size, a considerable distension of the abdominal parietes, and some impairment of health—all of which I consider favorable circumstances.

7. *The Existence or Non-Existence of Adhesions and Ascites.*

Of 41 cases of extensive adhesions, 25 or 60.97 per cent. recovered.
" 10 " slight " 7 or 70 " "
" 16 " no adhesions, 14 or 87½ " "

Still, I adopt the opinion of Dr. W. L. Atlee, that unless the adhesions are visceral or pelvic, they do not essentially increase the danger of the operation, if performed by an experienced operator.

Only 5 cases are noted of ascites as a complication, and of these 2 recovered, and 3 died. Very likely it did co-exist, however, in some of the cases in which tapping of the tumor was resorted to.

8. *Effects of previous Tappings on Ovariotomy.*—The following table shows the result of ovariotomy in 57 patients who had previously been tapped from 1 to 12 times:

| | Once tapped. | Twice tapped | 3 times | 4 times | 5 times. | 6 times | 8 times | 9 times | 12 times. |
|---|---|---|---|---|---|---|---|---|---|
| Recovered, ...... | 12 | 11 | 2 | 1 | 2 | 4 | 2 | 2 | 1 |
| Died, ............ | 2 | 10 | 2 | 1 | 2 | 2 | 1 | 0 | 0 |

Of the preceding 57 cases, 37, or 64.9 per cent. recovered. This is but 1.1 per cent. below the general average. The recoveries of those who had been but once tapped, amounted to 85.71 per cent. even. I have always regarded a single tapping as favorable on the whole, since it generally implies health slightly impaired, and the other advantages mentioned under a preceding head. Repeated tappings, on the contrary, imply much exhaustion in most cases; though not in the three above mentioned as 9 times, and 12 times tapped.

II. The Manner in which the Operation is Performed.—I shall consider, under this head, only the manner in which the pedicle of the tumor was managed, and the question whether, before closing the incision, the peritoneal cavity was sponged out, and whether the peritoneum was included by the sutures closing it.

1. *Management of the Pedicle.*—I here distinguish two classes of cases.

*A.* Those in which the *pedicle was left in situ*—whether after applying the double ligature, (Dr. C. Clay's method,) and which is brought through the lower end of the incision; or, after the ligature is cut close, (Dr. T. Smith's method.)

*B.* Those in which the *pedicle is kept projecting externally through the incision*, being maintained in that position either by sutures, needles, or the clamp.

Of 14 cases of the 1st class, 9 or 64.28 per cent. recovered.
" 107 " " 2d " 75 or 70.09 " "

It may, however, be added that the cases in which the clamp was applied were, most of them, operated upon by experienced operators, especially by I. B. Brown and T. S. Wells. Recent facts lead me to conclude, however, that Dr. T. Smith's method will prove to be the best.

2. *Was the Peritoneal Cavity sponged out before closing the Incision?*—I have not included the data for answering this question in my table; but I find (omitting all the cases in which there was no fluid to be removed from the peritoneal cavity) that—

| | | | | | |
|---|---|---|---|---|---|
| Of 50 cases in which it was carefully removed, | 35 or 70 | per cent. | recovered. |
| " 18 " " " not removed, | 10 or 55½ | " | " |

3. The data for answering the inquiry *whether the peritoneum was included by the sutures which closed the incision*, are also here omitted. I found that—

| | | | |
|---|---|---|---|
| Of 38 cases in which the sutures or needles were passed through the peritoneum, | 27 or 71.05 | per cent. | recovered. |
| Of 31 cases in which the peritoneum was *not* included, . . . . . . | 23 " 74.19 | " | " |

The causes of death, in the 11 fatal cases out of the 38, were not such as to be referred to the penetration of the peritoneum; and I think T. S. Wells' reasons for including that membrane quite conclusive.

III. The after Symptoms.—Many of the successful cases will be noticed as not having presented a single bad symptom after the operation. It cannot, however, be inferred from the non-appearance of bad symptoms during the first two or three days that the case will recover. The probability of peritonitis is thus diminished, but not necessarily that of septicæmia or of exhaustion.

IV. The after Treatment.—The data on this subject are also omitted in the present statistics. I found the custom of giving powerful doses of opiates after the operation, on the decline in the last half of the quadrennial period; and my conclusion on that point is, *give just opiates enough to allay pain, as it may rise, and to secure sleep, and no more.*"

The management of the pedicle is perhaps the one point of special importance in making this operation. What is the safest and best disposition to be made of it is a question which is not yet settled, and before positively deciding more observation is necessary. It has been disposed of in various ways and recoveries followed nearly all the different methods, but the best and safest disposition which can be made of it, is, perhaps, not yet determined. In what manner shall it be ligated, with what material, and what disposition made of the ends of the ligatures after they have been applied, are questions of great importance. Inclosing the pedicle in the incision with ligature or clamp outside the surface has been chosen by operators in a large proportion of cases, but objections to this plan are obvious. It may prevent or retard union; it is liable to cause traction upon the pedicle, places it in unnatural position, is sometimes with difficulty retained, and slides back to its place as the fastenings slowly give way. Returning the pedicle to the abdominal cavity with the ligatures hanging from the lower portion of the incision or with the ligatures cut short are two other ways which have been adopted, and recoveries have followed both these plans of treatment, but every surgeon can see some objections to both these methods. The ligature has been made double and tied both ways, or single, enveloping the whole mass. If the pedicle is thus treated there must be left in the abdominal cavity a mass of foreign material to be disposed of in some way. It must decay and discharge, or absorb and be thus removed, or become inert by being covered with a cyst, as other foreign bodies sometimes are. In the great proportion of cases the pedicle thus ligated will cause suppuration, and the mass thus strangulated will be dislodged with the usual products of inflammation. There are some objections to all plans yet devised, but doubtless there is great choice, and time and more extensive observation will aid in making selection.

It will be observed that another plan in this instance was adopted, and that the vessels of the pedicle were ligated separately, ligatures of silver and silk cut closely, and the hemorrhage having entirely ceased, the mass was allowed to return to its natural position. The philosophy was this: that the ends of the vessels with the small ligatures cut closely would be less productive of inflammation and suppuration, less liable to be followed by septicæmia, (pyæmia,) or other accidents known to be common causes of failure. The pedicle, however, did suppurate, and probably thus was discharged the small ligatures and the ends of enclosed vessels which it was expected might be absorbed, or where silver was used, become encysted and remain inert. Philosophy does not always agree with fact, nor yet one case establish a rule. We may be able in future to show that if the vessels of the pedicle are ligated separately and safely, that suppuration will not generally result, and that the divided pedicle will heal kindly, with little if anything to be either covered or removed. Though the results of this operation show nothing conclusive in favor of this manner of ligating the vessels, still when ligating separately can be practiced, it must have advantages over other methods, and is worthy of trial, unless it becomes apparent that hemorrhage is more liable to follow it, than in other modes of securing the vessels.

I have not the data to determine how the pedicle was treated in the 450 cases collected by Mr. Clay of Birmingham, but so far as I am aware this is the first case of ovariotomy treated in this manner. The principle, however, is the same, and perhaps the suggestion was first made by Dr. H. R. Storer of Boston, who removed successfully in September last, the uterus and both ovaries by abdominal section. The excision was made with the ecraseur, and six vessels were ligated with silver wire, other vessels were twisted, and three hours allowed to elapse from the commencement of the operation before the external wound was closed. During this period the pelvic cavity was repeatedly emptied of blood by sponging, but the hemorrhage was in this manner completely arrested.

There are many points of great practical importance in connection with the subject of removal of tumors by abdominal section, and many differences of opinion may be for the present expected. It may, however, be regarded as very well established that such operations are justifiable under certain circumstances, and it appears probable that there are fewer instances in which they should not be made than has been supposed, even by those most in their favor; they are operations in which the patients have everything to gain, and very little, if anything, to lose. Dr. Storer expresses his creed, as to abdominal sections, in the following general formulæ, which we quote entire, believing it a very good and safe "platform":

"1. Almost all ovarian tumors, a far greater majority than has been generally supposed, may be safely removed by abdominal section.

2. A certain proportion, as yet not ascertained, of uterine tumors, fibroid or fibro-cystic, may be safely removed in similar manner.

3. A large proportion of the fatal instances of either operation referred to, may be traced to neglect of simple precautions, prophylactic, immediate or subsequent.

4. Others still, to the fact that the patient was allowed to linger without assistance, till she was practically morribund, before commencement of the operation; and

5. Still others, that the surgeon's heart failed him after the abdomen had been opened, and the operation was not completed."

"Your duty and mine," says West, "is not in apathetic indifference, doing nothing, trying nothing, for a patient's cure, because her disease is one which hitherto has proved almost invariably mortal; but rather patiently, carefully, with much mistrust of our powers, much watchful scrutiny of our own motives, to apply ourselves to the trial of every means by which suffering may be mitigated or life prolonged. To this our common humanity prompts, our obligations as medical men compel us. It is to misinterpret both very grievously, if we merely content ourselves with doing nothing, but take shelter under noisy censure of the conduct, and uncharitable construction of the motives of those who read their duty differently."

Having decided the question that operation is proper and desirable, the following steps in the general plan of procedure may perhaps be as safely taken as any which have been proposed for the removal of these diseases. In the first place we should endeavor to make out the nature and extent of disease so far as possible—to determine that it is ovarian or uterine, cystic or fibrous, malignant or benign, to do this for obvious reasons, even though it be conceded that whatever the origin and character, removal offers the only rational hope of cure. Results thus far hardly justify removal by abdominal section of malignant disease; the same almost might be said of its removal by operation under any circumstances, the final result being ultimately about the same. If a tumor should prove to be of this character, no great harm would be produced—nothing very valuable lost; for the victim of such a malady to die, is gain. But I am not quite ready to despair of all hope, and would much rather err upon the side of removing even a malignant tumor, than of allowing a patient to die from the effects of a benign one, when removal offered chances of recovery.

The feasibility of operation being decided. it should be arranged with some thought and care. The condition of general health, regularity of the bowels, time of menstruation, etc., etc., require attention. Place for operation, nursing and means for proper after-treatment also depend upon the direction of the surgeon. Of the operation itself very little need be said. Anæsthesia is indispensable, not only to relieve pain but to avoid shock. Incision should be as short as will possibly admit of the elevation of the tumor, and the tumor should be lessened, when cystic, by evacuation of the contents of all the sacs. As to the treatment of the pedicle, nothing additional need be said. I am very well satisfied with the manner of treating it in the operation related, but I do not propose to say that it is the best, nor that when opportunity again presents I shall adopt it without modification, though we "speak well of a bridge which carries us safely," and I know of no better method in the management of the pedicle than the one selected. I have bestowed much thought upon the subject since my last operation, and I am led to believe that if I was to apply ligature to the pedicle as a whole, I would carry the extremities out through the vaginal septum, and in that way insure a drain for any purulent effusion which might take place. If I found that my ligatures applied to the vessels only, controlled the hemorrhage, and did not appear liable to slip or in any manner give way, I would by preference repeat the same operation as in the instance related.

Sponging the cavity of the abdomen, and handling and exposure of intestine and omentum should be avoided as far as possible; short incision greatly aids in this.

In closing the wound metallic sutures perhaps are best and should be used; they should be introduced down to the peritoneal membrane, perhaps it is safer to say through it, but it is difficult to see that it is not just as well to pass accurately down to it and avoid puncturing the membrane itself, but this point perhaps may remain undecided for the present. A wetted compress should be placed over the abdomen and retained by a bandage comfortably applied.

Pain should be allayed with anodynes, but the brain should not be too much oppressed with opiates. The urine should be frequently drawn, the bowels moved occasionally, perhaps by injections, and the general system sustained by nutritious food. All these conditions having been faithfully secured, there is great hope for those suffering from uterine or ovarian tumor.

